# Current Evidence about Nutrition Support in Cardiac Surgery Patients—What Do We Know?

**DOI:** 10.3390/nu10050597

**Published:** 2018-05-11

**Authors:** Aileen Hill, Ekaterina Nesterova, Vladimir Lomivorotov, Sergey Efremov, Andreas Goetzenich, Carina Benstoem, Mikhail Zamyatin, Michael Chourdakis, Daren Heyland, Christian Stoppe

**Affiliations:** 1 Department of Intensive Care Medicine, University Hospital RWTH, D-52074 Aachen, Germany; cbenstoem@ukaachen.de; 2 3CARE—Cardiovascular Critical Care & Anesthesia Evaluation and Research, D-52074 Aachen, Germany; andreas@goetzenich.net; 3 Department of Anesthesiology and Intensive Care Medicine, National Pirogov Medical Center, 105203 Moscow, Russia; d10001letopisec@mail.ru (E.N.); zamyatinmn@pirogov-center.ru (M.Z.); 4 Department of Anesthesiology and Intensive Care Medicine, E. Meshalkin National Medical Research Center, 630055 Novosibirsk, Russia; v.lomivorotov@gmail.com (V.L.); sergefremov@mail.ru (S.E.); 5 Department of Thoracic, Cardiac and Vascular Surgery, University Hospital RWTH, D-52074 Aachen, Germany; 6 Department of Medicine, School of Health Sciences, 54124 Thessaloniki, Greece; mhourd@gapps.auth.gr; 7 Clinical Evaluation Research Unit, Kingston General Hospital, Kingston, ON K7L 2V7, Canada; dkh2@queensu.ca

**Keywords:** cardiac surgery, cardiopulmonary bypass, systemic inflammatory response, nutrition risk stratification, underfeeding, postoperative nutritional management, supplemental parenteral nutrition, enteral nutrition, pharmaconutrition

## Abstract

Nutrition support is increasingly recognized as a clinically relevant aspect of the intensive care treatment of cardiac surgery patients. However, evidence from adequate large-scale studies evaluating its clinical significance for patients’ mid- to long-term outcome remains sparse. Considering nutrition support as a key component in the perioperative treatment of these critically ill patients led us to review and discuss our understanding of the metabolic response to the inflammatory burst induced by cardiac surgery. In addition, we discuss how to identify patients who may benefit from nutrition therapy, when to start nutritional interventions, present evidence about the use of enteral and parenteral nutrition and the potential role of pharmaconutrition in cardiac surgery patients. Although the clinical setting of cardiac surgery provides advantages due to its scheduled insult and predictable inflammatory response, researchers and clinicians face lack of evidence and several limitations in the clinical routine, which are critically considered and discussed in this paper.

## 1. Introduction: Cardiac Surgery, Inflammation and Current Standard of Nutrition

Patients undergoing cardiac surgery regularly experience a systemic inflammatory response, which contributes to acute and persistent organ injury. With an increasingly older population undergoing increasingly complex cardiosurgical procedures, the incidence of comorbidities and malnutrition rises. The nutritional status and adequate nutrition therapy are crucial factors contributing to the outcome of patients. The following sections will give a basic background on the inflammatory reaction expected after cardiac surgery, and will illustrate the pathomechanisms of organ dysfunctions and their influence on clinical outcome. In extension, we summarize current nutritional practice, which is thought to influence outcomes and we will illuminate opportunities for improvement in every day clinical practice.

### 1.1. Inflammation in Cardiac Surgery

**Figure 1:**
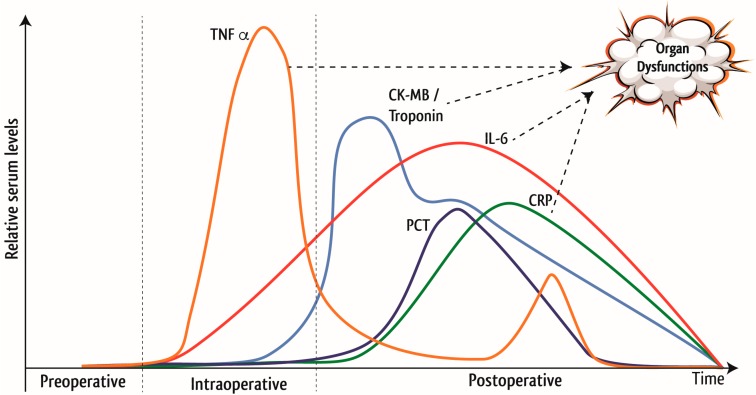
Time course of inflammation and development of organ dysfunction in cardiac surgery. Cardiac surgery leads to an anticipated rise of inflammatory mediators, such as TNFα, IL-6, procalcitonin (PCT), c-reactive protein (CRP) and markers of myocardial damage, such as creatine kinase (muscle/brain) (CK-MB) and troponin [11]. These mediators trigger the development of postoperative organ injuries, which are explained in greater detail in Figure 2.

Patients undergoing cardiac surgery experience a complex systemic inflammatory response syndrome, which manifests as pyrexia, tachycardia, leukocytosis, hypotension, edema, and organ failure. Several stimuli lead to systemic inflammation reactions during and after cardiac surgery. The surgical trauma induces the activation of neutrophils, endothelial cells and platelets and the release of mediators of the inflammatory response, such as tumor necrosis factor α (TNFα) and diverse interleukins (IL) [1,2]. The foreign surface contact during cardiopulmonary bypass (CPB) leads to the activation of cellular components such as leukocytes and platelets [3,4] and activates further humoral mediators [5], such as the complement system [6,7,8], as well as kallikrein cascades, inducing a release of inflammatory mediators such as TNFα, IL-1, IL-6 and IL-8 (see Figure 1) [9,10].

**Figure 2:**
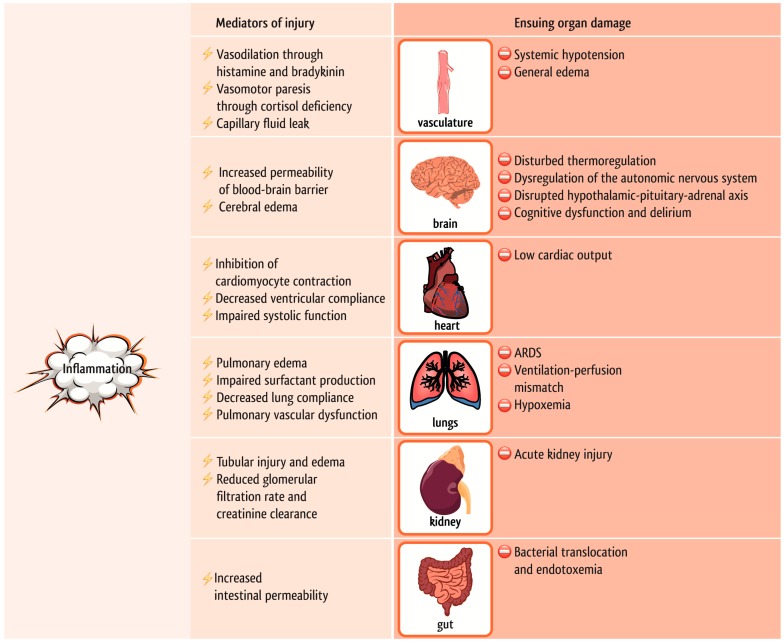
Effects of inflammation on different organs.

After an ischemic period during the cross-clamping of the aorta, the reoxygenation of the tissues further triggers the inflammatory response. This ischemia-and-reperfusion (I/R)-injury can also be divided into leukocyte-dependent-mechanisms—through the interaction of neutrophils and endothelial cells—and non-leukocyte-dependent pathways, such as the release of reactive oxygen species, arachidonic-acid metabolites and cytokines, as well as increased nuclear factor kappa-light-chain enhancer of activated B cells (NFκB) activity [1,9,12,13]. Hemodilution through large extracorporeal circuits and blood loss during surgery often create a need for blood transfusions, which may further trigger inflammatory reactions [13,14,15]. Notably, the so called enteral hypoperfusion during cardiac surgery increases the permeability of the gut mucosae and the transferal of intestinal bacteria into the bloodstream. Bacterial lipopolysaccharides from gram-negative bacteria may further induce TNFα and IL-6 production, complement activation, and the release of cytokines and nitric oxide, which further increase the extent of organ dysfunctions (Figure 2, for comprehensive review please see: [13]).

The most frequent organ dysfunctions after cardiac surgery are pulmonary dysfunction (up to 79%) [16], ventricular dysfunction (up to 70%) [17,18], postoperative cognitive dysfunction (up to 65%) [19,20], and acute kidney injury in a third of all patients [21]. In particular, high-risk cardiac patients with extended surgical procedures and durations of CPB are exposed to a significantly higher inflammatory response with harmful effects. For this reason, various clinical trials have attempted to reduce the perioperative inflammatory response by administration of different immune-modulatory agents, which are outlined in Section 4.

Organ dysfunctions cause a prolonged need of life-sustaining therapies and consequently a longer stay on the intensive care unit (ICU). The influence of nutritional deficiencies on these pathomechanisms are described in the next section.

### 1.2. Importance of Nutrition in Cardiac Surgery

**Figure 3:**
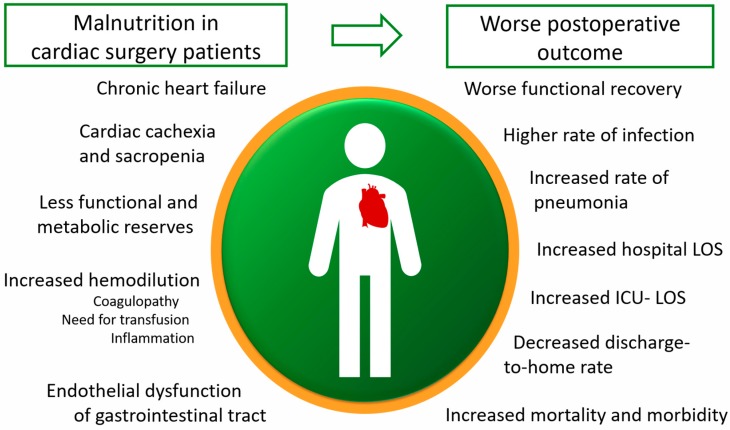
Influence of malnutrition on the outcome of cardiac surgery patients.

Malnutrition is a subacute or chronic state of disordered nutrition in which a combination of varying degrees of over- or undernutrition and inflammatory activity have led to a change in body composition and diminished function [22,23], or more simply, as nutrition imbalance [24]. Cardiac surgery patients who are malnourished prior to surgery have been demonstrated to show worse outcomes after surgery, including higher morbidity and mortality as summarized in Figure 3 [25,26].

Nutritional deficiencies contribute to the pathomechanisms of inflammation due to reduced defense mechanisms and depleted metabolic reserves in the patient. Malnourished patients are more susceptible to the surgical trauma, I/R-injury, anesthesia-related complications, hemodilution, as well as inflammation. Malnutrition promotes endothelial dysfunction of the gut mucosa, allowing for a possible bacterial translocation [27]. A preexisting malnutrition is further aggravated by preoperative fasting and the commonly observed postoperative delay of nutrition support. In this context, numerous observational studies demonstrated a significant depletion of macro- and micronutrients, as well as the importance of energy and protein metabolism in the early stage after cardiac surgery [25,26]. In the same vein, Drover et al. demonstrated in a retrospective analysis that patients after surgery are at an increased risk of malnutrition during the postoperative ICU stay [28]. In summary, nutrition support improves the recovery of patients in various ways [29,30]:

- Maintenance of metabolism
- Attenuation of catabolism
- Maintenance of gut-integrity
- Reduction of postoperative complications
- Improved wound healing
- Securing adequate hydration and euglycemia.

### 1.3. Current Nutritional Practice: Systematic Review, Guidelines and Observational Data

#### 1.3.1. Systematic Review

Until today, evidence of well-designed, well-powered randomized controlled trials investigating the clinical significance of an early initiated nutrition therapy in high-risk cardiac patients after surgery remains sparse. Therefore, we conducted a systematic literature review focusing on nutritional protocols e.g., hypocaloric feeding or amount of macronutrients in critical illness with special focus on patients after cardiac surgery. The PICO-framework—an acronym for participants, intervention, comparison and outcome—addresses essential aspects of a well-built clinical research questions. It was used to define the focus of this systematic literature review. Studies were included if they met the following criteria:

(1) population: critically ill, adult patients after cardiac surgery; (2) intervention and comparison: any type of nutritional protocol, e.g., early intense versus early lower-dose enteral nutrition, early versus delayed enteral nutrition, enteral nutrition alone versus enteral nutrition plus supplemental parenteral nutrition; (3) any type of clinical outcome; (4) type of studies: prospective or retrospective observational studies, randomized or non-randomized clinical trials, systematic reviews of literature and meta-analyses. Medical Subject Headings (MeSH) was applied to identify most appropriate search terms. Consequently, the following search strategy (example given for MEDLINE) was developed to identify matching studies: ((Nutrition Therapy [MeSH Major Topic]) AND (cardiac [Title/Abstract] OR heart [Title/Abstract])) AND surg* [Title/Abstract]. To locate relevant articles, six bibliographic databases (Cochrane Database of Systematic Reviews (CDSR), Cochrane Central Register of Controlled Trials (CENTRAL), Database of Abstracts of Reviews of Effects (DARES), and the Medical Literature Analysis and Retrieval System Online (MEDLINE) were searched. Importantly, no sufficiently designed, adequately powered, randomized controlled trials investigating the effect of nutritional therapy in cardiac patients after surgery were available. Yet, several small studies have provided initial evidence on the feasibility and clinical significance of nutritional therapy in cardiac surgery patients. Based on the above-described findings, we proceed with a narrative review of the literature to illuminate current evidence about nutrition support in cardiac patients relevant for daily clinical practice to demonstrate:

- The low evidence received from rather small studies with from heterogeneous patient populations with different nutrition interventions and non-comparable outcome assessments
- The resulting need for adequate studies and
- The urgent need for specific guidelines for this cohort of critically ill patients, as current clinical practice may lead to malnutrition in these patients.

#### 1.3.2. Current Nutritional Practices: Guidelines and Observational Data

**Figure 4:**
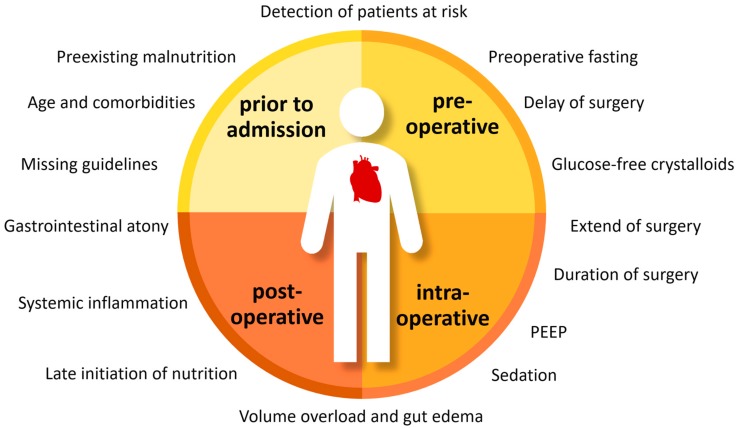
Extrinsic risk factors influencing nutrition support in cardiac surgery, PEEP: positive end-expiratory pressure.

In clinical practice, often little attention is paid to nutrition support and various extrinsic risk factors hinder the adequate supplementation of nutrients as summarized in Figure 4. Iatrogenic malnutrition is especially due to delayed initiation of nutrition support after cardiac surgery, but also due to an inadequate supplementation of protein and energy. The European Society for Clinical Nutrition and Metabolism (ESPEN) as well as the American Society for Clinical Nutrition and Metabolism (ASPEN) recommend the initiation of enteral nutrition within 24 h after surgery and a supplementation of 25–30 kcal and 1.5–2.5 g of protein per day and kilogram ideal body weight for critically ill patients [29,31]. Yet, besides these general guidelines for surgical patients, there exist no specific guidelines defining the perioperative nutrition support for cardiac surgery patients.

In a prospective observational study, Rahman et al. recently demonstrated as analysis of an international nutrition survey in critical care units around the world that nutritional adequacy was low with respect to both energy and protein supplementation in cardiac surgery patients [32]. In addition, they confirmed that patients undergoing cardiovascular surgery were at the highest risk for iatrogenic malnutrition due to withholding of nutrition support during the early postoperative course. In a study including 787 patients cardiac surgery patients with an ICU stay of greater than 3 days, the authors found that 40% of patients received no nutrition support at all and the mean time from ICU admission to initiation of enteral nutrition (EN) was 2.3 ± 1.8 days [32]. With EN alone, as well as with combined parenteral nutrition (PN), patients received less than a third of calories and protein as shown in Table 1. Furthermore, patients with later initiation of nutrition support have even lower total nutritional adequacy than other surgical or medical ICU patients, indicating the need to improve nutrition practice in that population [28].

Nutrition adequacy was defined as the total amount of calories or protein received from EN and propofol, as well as PN, divided by the amount prescribed as per the baseline assessment and expressed as a percentage.

Up to 8% loss of body weight and a consistently positive nutrition risk screening (NRS 2002) were reported in patients scheduled for cardiovascular rehabilitation for 1–6 months after treatment for ischemic, valvular, or combined causes of heart diseases [33].

Given these findings, we can conclude that nutritional adequacy in cardiac surgery patients is low with respect to both energy and protein intake. However, in this observational study by Rahman et al., an improved nutritional adequacy was not associated with reduced overall mortality in all cardiac surgery patients per se. Given these findings, further research is needed to identify those cardiac surgery patients, who are most likely to benefit from an intense nutrition support.

## 2. Nutrition Screening in Cardiac Surgery Patients

Detecting patients at high nutritional risk is essential for an adequate therapy. Nutritional status assessment scores are recommended by current international nutrition guidelines but are not validated for this population and rarely used in cardiac surgery. Using the well-established nutrition screening tools Short Nutritional Assessment Questionnaire (SNAQ), Malnutrition Universal Screening Tool (MUST), Malnutrition Screening Tool (MST) Nutrition Risk Screening 2002 (NRS-2002), Mini Nutritional Assessment Short-Form (MNA-SF) and Subjective Global Assessment (SGA), which are described in detail in Table 2, Lomivorotov et al. found that depending on the used nutrition screening tool, the percentage of malnourished cardiac surgery patients prior to surgery ranged between 4.6–19.1% [25]. Besides, the investigators demonstrated that the majority of these nutrition screening tools are insufficiently sensitive to the risk of developing postoperative complications, whereas the reasons for these findings probably result from the different pathophysiology of postoperative cardiac surgery patients compared to other critically ill ICU patients.

As current findings indicate high malnourishment rates in cardiac patients [52,53], it is crucial to consider the patients’ nutritional profiles preoperatively and to simultaneously devote further attention to the conception of individual diets for preoperative optimization in these patients [26]. Thus, the assessment of preoperative nutritional status may guide health care professionals to consider early nutrition interventions prior to surgery in patients at high risk of developing postoperative complications [54]. In addition, cautious attention must be paid on postoperatively assessed reliable outcome variables, which are summarized in Table 3.

In clinical practice, metabolic control, maintenance of normoglycemia, substitution of electrolytes according to blood work remain the short-term goals for nutrition support. The nutrition support has to be adapted to the individual tolerance of each patient. Physical examination, evidence of gastrointestinal motility, absence of diarrhea, vomiting and abdominal complaints may serve as parameters to evaluate the tolerance of nutrition [29,31,55].

There are no valid biochemical or anthropometrical parameters recommended by current guidelines. Frequently used parameters, such as weight, nitrogen-balance, serum-albumin, handgrip-strength, CRP, transferrin and circumference of extremities did not show any influence on patient-outcome [31,56,57]. Studies evaluating the relevance of body mass index (BMI), albumin and prealbumin levels demonstrated that these are independent predictors of morbidity and mortality after coronary artery bypass graft (CABG) and valve surgeries [58,59]. Furthermore, low prealbumin levels provide incremental information compared with BMI and albumin and were associated with prolonged duration of ventilation and increased incidence of postoperative infections [60]. However, the validity of serum albumin, prealbumin and BMI calculation using “dry” weight needs further validation as a way of identifying malnourished patients before surgery, as for example albumin has a turnover time of 20 days and its serum level is influenced by numerous factors. Yet, it is still recommended to be used as a component of the preoperative nutrition screening until a better marker is available.

## 3. Perioperative Nutrition Support in Cardiac Surgery Patients

### 3.1. Preoperative Nutritional Optimization in Cardiac Surgery Patients

Although the inflammatory response to cardiac surgery shares mechanisms with that observed in septic patients, the postsurgical inflammatory response is more predictable, mainly featuring the release of pro-inflammatory markers and reactive oxygen species. Therefore, the pre-operative period may represent an attractive time window in which to optimize nutritional status, correct deficiencies, and enhance immune defense mechanisms before surgery. This period is an especially effective time to act upon modifiable risk factors and potentially lower the risk of intra- and postoperative complications. The bulk of the literature on perioperative optimization in heart failure patients comes from anesthesiology and hence focuses on intra- and immediate postoperative management, when it may be too late to intervene and alter the outcome of a patient entering the operating room in a decompensated state [61]. Interestingly, guidelines on the cardiovascular evaluation and management of patients prior to non-cardiac surgery are available, but no comparable recommendations have been published concerning cardiac surgery [61,62], which is probably because the patients’ outcome was thought to be mainly influenced by the surgical procedure itself.

In cardiac surgery patients with progressive heart failure, preoperative fasting further (i) aggravates commonly observed symptoms such as dyspnea, with a resulting increase in work of breathing, and (ii) it may worsen gut edema as well as (iii) hepatic congestion, which may further result in early satiety and nausea [63]. The chronic inflammatory state and the metabolic disturbances induced by chronic inflammation are shared by all disease-induced cachectic processes, including cancer, chronic obstructive pulmonary disease and advanced heart failure [64,65]. Clinically, this state is characterized by protein-calorie malnutrition, with systemic manifestations of lassitude, weakness, and poor wound healing, leading to frailty and significant comorbidities.

**Figure 5:**
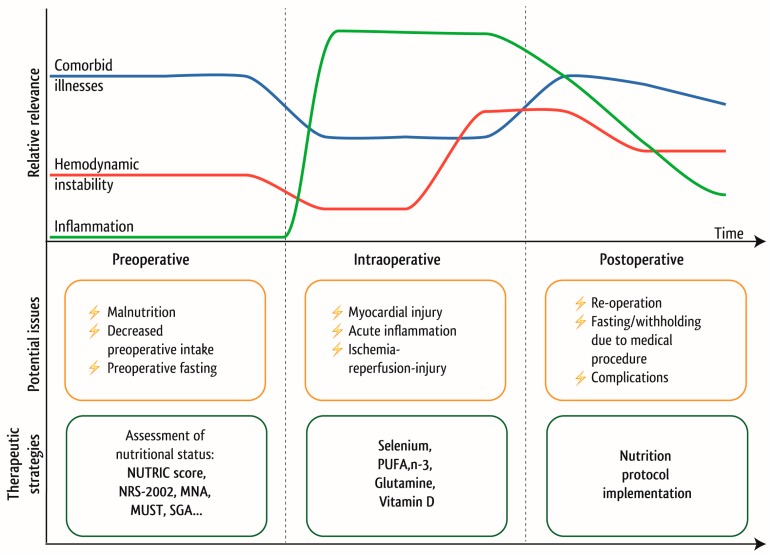
Possible areas of interest to optimize the nutritional status depending on the stages of hospitalization.

As it is generally accepted that preoperative medical and nutritional optimization is necessary and may provide beneficial effects if performed in patients scheduled for major surgery, multimodal approaches, such as enhanced recovery after surgery (ERAS) programs, may be useful in cardiac surgery patients to reduce surgical stress, maintain physiological functional capacity, and facilitate postoperative recovery by providing the best available evidence. However, while there several approaches available for other types of major surgery [66,67,68], evidence is lacking on how the principles of ERAS could be applied to cardiac surgery [69]. Further confirmation of the importance of a preoperative nutrition intervention is necessary. Besides, it must be acknowledged that a pre-operative nutrition risk assessment and timely intervention is hindered by logistical difficulties, as more than half of patients who undergo cardiac surgery are admitted as outpatients within 12–24 h before surgery. Clinicians will need to overcome this problem and consider an interdisciplinary outpatient-approach to optimize the nutritional status prior to patient’s admission in collaboration with surgeons, cardiologists and general practitioners. The potential areas of interest linked to therapeutic strategies to optimize nutrition practice are outlined in Figure 5.

### 3.2. Postoperative Nutrition Support in Cardiac Surgery Patients

While various large-scale randomized controlled studies evaluated different post-operative nutrition strategies in rather mixed cohorts of critically ill patients, only few small clinical studies specifically investigated its effects in cardiac surgery patients. In these studies, malnutrition has been reported to increase morbidity and mortality after cardiac surgery [70,71], as well as it may reduce the muscle mass of the left ventricle. Some cardiac surgery patients experience a complicated postoperative course, requiring pharmacological and/or mechanical cardiac support, as well as prolonged mechanical ventilation. These patients are frequently hypercatabolic, unable to feed themselves for more than 5–6 days and are in special need of intense nutrition support [72,73]. Besides, it was demonstrated that weight-loss in patients discharged after cardiac surgery was accompanied by a persistent inflammatory response resulting in decreased physical functioning [74]. However, most cardiac surgery patients stay briefly in the ICU and can resume oral feeding within 1–2 days after surgery, hence, they do not require an intense nutrition support.

Visser et al. studied the effect of perioperative nutrition in cardiac surgery on the myocardial inflammatory response, supplementing either no nutrition, EN or PN from 2 days before to 2 days after CABG. While both forms of nutrition contained comparable macro- and micronutrients, myocardial atrial tissue samples before and after revascularization demonstrated no significant differences in the myocardial inflammatory response [75].

The recent CoCoS trial evaluated the influence of nutrition therapy on possible alterations in caloric deficit, morbidity and mortality. No significant differences in patients, laboratory or mortality profile between the intervention group, which received intense nutrition support, and a retrospective control group were found. However, there were significantly less arrhythmias (7% versus 31%; *p* = 0.0056), and significantly less pneumoniae (7% versus 22%; *p* = 0.0183) in male intervention-group-patients receiving combined CABG and aortic valve surgery. In addition, survival was significantly higher in female patients receiving intense nutrition support than in the control group for both CABG (100% versus 83%; *p* = 0.0015) and aortic valve surgery (97% versus 78%; *p* = 0.0337) [76].

The data derived from this trial support the hypothesis, that patients with either high nutrition risk or at elevated risk for prolonged ICU stay are the patient groups which will most likely benefit the most from a nutrition intervention and to determine the effect of prolonged EN on patients’ clinical outcome. Despite well-established scoring systems for perioperative risk stratification, it is still challenging to identify patients at high nutrition risk early during their postoperative course, which may enable to start early an adequate nutrition support for these patients.

#### 3.2.1. Enteral Nutrition

The role of postoperative nutrition support is to maintain nutritional status and energy requirements in the catabolic period after surgery. An interruption of nutritional intake is frequently observed after surgery, although it is evident that early oral and/or enteral food intake is possible, diminishes the risk of infectious complications and favors shorter hospital stays [77,78,79]. Therefore, early nutrition is encouraged by international nutrition societies to enhance recovery after surgery [66,67,68]. While the function of the gastrointestinal (GI) tract is the main determinant for initiation of EN after abdominal surgery, the key factor for initiation of nutrition in cardiac surgery patients may be hemodynamic stability, as the recently revised ASPEN guidelines recommend that EN should be withheld until the patient is hemodynamically stable [72].

Despite the lack of evidence, EN is commonly considered to be contraindicated as it may negatively affect gut integrity during a state of severe circulatory compromise in patients requiring high levels of vasopressor support, resulting in (1) alteration of splanchnic perfusion and (2) an increased risk of GI complications, such as bowel ischemia. In addition, there are relevant practical hurdles such as the numerous interruptions of enteral feeding, pyloric dysfunction and intestinal atony, which are frequently seen in patients after major surgical procedures. In this context, there are several studies examining the GI response to enteral nutrition in the presence of compromised hemodynamics and evaluating intestinal intolerance in cardiac surgery patients (Table 4).

In the prospective study of Berger et al., a mean energy delivery of 70 ± 35% of the target could be achieved via EN, even though most patients were on vasoactive drugs for many days [80]. Dopamine and norepinephrine were significantly negatively correlated with enteral feeding, while there was a negative trend with dobutamine, indicating that the initiation of EN lead to a significant reduction in vasoactive medications. No patient experienced any serious GI complications and EN was possible. Revelly et al. studied nine patients requiring hemodynamic support in their hemodynamic and metabolic reaction to the initiation of EN. Physiological hemodynamic and metabolic reactions as well as no serious GI complications were observed [81]. In a comparable manner, Kesek et al. started EN within 3 days in accordance to the patient’s needs, which were calculated by the Harris–Benedict equation [82]. The authors did not provide a detailed description or the duration and doses of vasoactive drugs. Diarrhea and gastric residual volumes were frequent; however, the clinical relevance remains unclear [83]. Clinically significant GI complications were notably infrequent, and the authors concluded that early EN could be safely initiated in the cardiac surgery intensive care population.

A study by Flordelís Lasierra et al. including cardiac surgery patients with hemodynamic failure (dependence on two or more vasoactive drugs and/or mechanical circulatory support), EN was supplemented with a mean energy delivery 1228.4 kcal/day over a mean of 12.3 days. The mean energy target was achieved in 15 patients (40.4%). The most common EN-related complication was constipation, whereas no case of mesenteric ischemia was detected, further supporting the feasibility and safety of EN in these patients [84].

Despite the small number of patients included in these studies and the differences among their inclusion and application strategies, it is important to note that enteral nutrition has repeatedly been demonstrated to be feasible and that the circulatory and metabolic response to EN is adequate during the early postoperative course after operation in patients with acute severe circulatory failure. Furthermore, these studies indicated a potential beneficial effect of enteral nutrition due to its ability to maintain the splanchnic perfusion, which is of particular importance for cardiac surgery patients with an increased risk for postoperative mesenteric ischemia. However, all the mentioned studies also concluded that it was not possible to meet the nutritional requirements with EN alone and suggested the addition of supplemental parenteral nutrition (sPN), which need to be systematically investigated in future studies.

#### 3.2.2. Parenteral Nutrition

As intestinal ischemia is a rare, but highly lethal complication after cardiac surgery, occurring in 0.5% and being fatal in up to a quarter of patients [85,86,87,88,89], the use of PN is often favored in cardiac surgery patients, especially within the first days after operation. However, there is no sufficient evidence available to evaluate the role of postoperative PN and its influence on clinically relevant outcome data, including survival, disease progression and morbidity in cardiac surgery patients. This lack of data may be due to the usually short duration of stays in the intensive care unit. Moreover, insufficient nutritional assessment prior to operation may prevent practitioners from starting parenteral nutrition in malnourished patients soon after surgery in accordance with actual guidelines.

PN may be used as sole nutrition or as sPN, as demonstrated almost 3 decades ago by Paccagnella et al. in 1994 [90], who examined the hemodynamic, metabolic, and nutritional response to nutrition support of patients with severe cardiac cachexia before and after major cardiac surgery. Patients were allowed to eat ad libitum, and sPN was then provided in order to achieve a maintenance level of nutrition support. The results suggested that this approach is both safe and effective.

Existing guidelines recommend the initiation of PN in all critically ill patients within 3–7 days after admission if EN is contraindicated or cannot be tolerated in patients with low nutrition risk and within 24 h in patients with high nutrition risk [29,31]. PN secures reaching energy and protein targets and avoids the potential complications of EN. Concerns regarding PN are the potential risk of overfeeding with hyperglycemia, elevated liver enzymes and increased rate of blood stream infections. Current evidence remains inconclusive, but there seems to be no difference regarding clinical outcome between EN and PN [91,92,93]. However, in the EPaNIC Trial of Casaer et al., a lower rate of infection was observed with a later achievement of caloric targets [94]. In any case, it is recommended to evaluate both provision as well as tolerance frequently and switch to the least invasive and most physiological route of administration of nutrition which is feasible for each individual patient.

Intravenous fish oil (FO)-based lipid emulsions (LEs) are of increasing interest as part of the parenteral nutrition support. FO is rich in ω-3 polyunsaturated fatty acids (ω-3-PUFAs), such as eicosapentanoic acid (EPA) and docosahexaenoic acid (DHA), which exhibit anti-inflammatory and immunomodulatory effects. Preliminary evidence received small phase II trials on FO-containing emulsions in cardiac surgery have provided preliminary evidence that preoperative FO infusion is a promising strategy to modulate the biological and clinical response to cardiac surgery with the use of CPB [95,96,97,98]. Various studies indicate that ω-3-PUFAs exert beneficial effects on the cardiovascular system that may ultimately reduce the risk of cardiac death and lower the incidence of perioperative atrial fibrillation (AF) in cardiac surgery, whereas current data on this topic are inconclusive, perhaps because of the different supplementation strategies and the dependence of the results on the type of surgical procedure [99]. Recently, Berger and colleagues demonstrated that three repetitive infusions of 0.2 g/kg FO emulsion, significantly increased PUFA concentrations in platelets and atrial tissue membranes within 12 h of the first FO administration and reduced the inflammatory response, whereas its clinical significance still remains largely speculative [96]. In this context, Christou et al. reviewed current trials in view of the role of ω-3 PUFA supplementation for prevention of AF after cardiac surgery. Here, the investigators observed conflicting results [100], which probably occurred for 2 main reasons: (1) most studies applied n-3 PUFA treatment only postoperatively [99]; (2) In the case of treatment before cardiac surgery, its duration was insufficient to result in adequate incorporation of n-3 PUFA in sarcolemmal myocardial membranes, leading to probably insufficient biological and clinical relevant effects [98,99,100,101,102,103]. Moreover, DHA treatment appears to be more efficient than EPA treatment in reducing the incidence of postoperative AF [102,104,105]. However, a meta-analysis including all 6 placebo-controlled randomized controlled trials (RCT) [95,101,106,107,108] found a regression rate of 0.92 (95% CI, 0.78–1.10) and could not detect a significant clinical relevant effect, as all included trials were limited by low statistical power. Notably, as no other relevant postoperative complications have been adequately evaluated, further studies need carefully to consider potential side effects. Furthermore, no data exists about its potential role in high-risk cardiac surgery patients, with complex surgical procedures, which are at increased risk for the development of postoperative complications. Given its outlined biological rationale and previously demonstrated beneficial effects in small clinical trials [95,96,97,98], following adequately designed studies focusing on functional outcomes are still needed to clarify the role of fish oil in these patients at high risk for the development of organ dysfunction. Regarding international recommendations, the ESPEN Expert Group currently supports the use of olive oil and FO in nutrition support in surgical and non-surgical ICU patients but considers that further research is required to provide a more robust evidence base [109].

## 4. Micronutrients in Cardiac Surgery Patients

### 4.1. Glutamine

One immune-active substance, the non-essential amino acid glutamine, is the most abundant amino acid in the human body and showed cardioprotective effects in several clinical trials. The perioperative administration of both parenteral (N(2)-L-alanyl-L-glutamine) [110] and enteral [111] forms of glutamine leads to reduced myocardial injury as assessed by reduced postoperative troponin I concentration among cardiac surgery patients.

However, in view of the insufficient evidence, recent guidelines state that routine supplementation with glutamine cannot be recommended due to the unproved clinical benefits in cardiac surgery patients and even a risk of harm, which has been demonstrated in critically ill patients [31].

### 4.2. Selenium

Selenium is a trace element that is important for many of the body’s regulatory and metabolic functions, especially during times of stress [112,113]. In an observational study, the majority of patients undergoing cardiac surgery exhibited a significant selenium deficiency prior to CPB, which was further aggravated with increasing CPB time, leading to an insufficient capacity to withstand the stress of surgery [113]. In a subsequent non-randomized interventional trial, a high-dose selenium supplementation was effective in preventing this decrease of intraoperative circulating selenium levels and clinical outcomes were superior in this supplemented group compared with a historical control group [114]. Recently, a randomized controlled study demonstrated the safety and feasibility of high-dose selenium supplementation (4000 µg) in cardiac surgery patients, whereas no significant clinical effects could be detected [115]. In view of these data, a large-scale multicenter trial is currently being performed to evaluate the clinical significance of high-dose (2000 µg) perioperative sodium selenite supplementation in patients at high risk after cardiac surgery [116].

### 4.3. Vitamins

Few data are available regarding vitamin supplementation in cardiac surgery patients. Among the most relevant vitamins, thiamine and vitamins D and C are the most promising candidates and have been studied in several trials. Thiamine, the essential co-factor for pyruvate dehydrogenase function, is responsible for adequate aerobic metabolism. Preliminary studies demonstrated that thiamine levels are decreased after cardiac surgery and that low serum levels are inversely associated with blood lactate level [117,118], which, in turn, predicts postoperative mortality and morbidity [119,120]. However, recently published RCTs did not support the hypothesis that thiamine administration during cardiac surgery decreases postoperative blood lactate levels and improves clinical outcomes and therefore cannot be recommend based on current evidence [121,122].

Vitamin D is known to affect the bones, the muscles, the blood vessels, cell proliferation and differentiation, autoimmune processes and the immune system in parallel with the regulation of calcium homeostasis [123]. Therefore, vitamin D deficiency leads to skeletal and non-skeletal diseases and is associated with various respiratory, immune, infectious, neurological and cardiovascular diseases. It is involved in numerous physiological mechanisms desirable for cardiac surgery patients, such as regulation of arterial stiffness and endothelial function [123]. However, in one retrospective study, low vitamin D concentrations before surgery were not associated with increased mortality and morbidity, while the significance of intraoperative changes and potential differences between the biological active (1, 25OH) and inactive form (25OH) remained unknown [124]. Consequently, further research is needed to clarify the clinical significance of Vitamin D for cardiac surgery patients.

Vitamin C shows pleiotropic functions in the human biology and reduced oxidative damage and resulting organ injury in critically ill patients with sepsis or septic shock [125]. In cardiac surgery patients, preliminary studies indicate a beneficial effect of Vitamin C supplementation on the occurrence of postoperative outcome [126]. Besides, a recent meta-analysis of small preliminary studies demonstrated that administration of vitamin C is effective as prophylaxis for prevention of postoperative AF [127]. Yet, no observational data exists, which carefully investigated the circulating Vitamin C levels in the blood. Adequately designed studies are now encouraged to comprehensively investigate the effect of an appropriate Vitamin C supplementing strategy on the patients’ inflammatory response and to evaluate its clinical effects on patients’ mid- to long-term outcomes.

## 5. Actual Recommendations and Road Map for Future Research Activities

**Figure 6:**
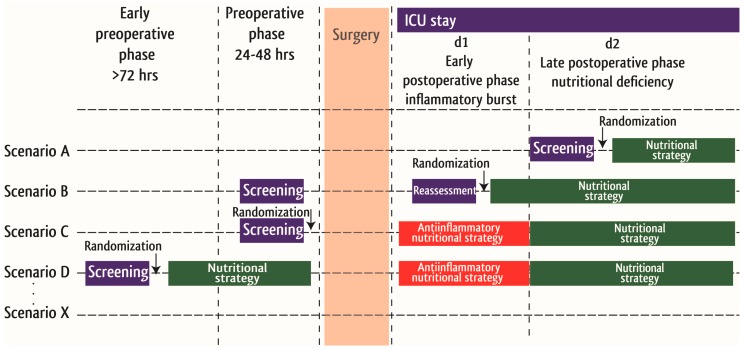
Scheme for future studies evaluation the clinical significance of perioperative nutrition support in cardiac surgery patients.

The systematic review of the literature already demonstrated the need for well-designed and adequately powered studies to develop specific evidence-based recommendations and guidelines for this cohort of critically ill patients. As cardiac surgery itself is a scheduled insult, it offers opportunities for nutrition support through the preoperative, intraoperative and postoperative window. To comprehensively address the clinical relevance of nutrition support in cardiac surgery patients we have developed with cardiac surgeons, cardiologist dieticians and the following research strategies, which should be the base for future clinical studies in this field to improve the nutrition support for this group of critically ill patients, as outlined in Figure 6.

In addition, more research is warranted to evaluate the effect of an intense nutrition support on functional outcomes in this cohort of critically ill patients and to focus on identification of patients, which may benefit from an intense nutrition support.

In absence of specific guidelines for the nutrition support of cardiac surgery patients, actual guidelines for the postoperative nutrition support of surgical and critically ill patients [29,31] should be considered in the clinical practice until more specific recommendations—based on specific, adequately designed studies—are available. In addition to these guidelines—based on current evidence received from preliminary results—the following aspect should be considered in the treatment of cardiac surgery ICU patients:

- Assessment of preoperative nutritional status may guide health care professionals to consider early preoperative nutrition interventions in patients at elevated risk of developing postoperative complications
- Postoperative oral/enteral nutrition intake should be resumed early in the hemodynamic stable cardiac surgery patients
- Cardiac surgery patients with brief ICU stays often recover within the first 1–2 days after surgery and are considered less likely to benefit from an intense nutrition support
- Intense nutrition support with adequate caloric and protein intake is of special relevance for patients with prolonged ICU stay or patients at high nutritional risk
- Clinical significance of pharmaconutrients, fish oil and vitamins need further clarification in adequately designed clinical studies

## 6. Conclusions

Despite substantial procedural advances, open-heart surgery continues to be associated with disconcerting complication rates, often necessitating a prolonged ICU stay until the organ functions recover, especially in high-risk cardiac surgery patients with significant comorbidities and complex cardiac surgical procedures. While cardiac surgery patients with brief ICU stay are considered less likely to benefit from an intense nutrition support, an early intense nutrition support should be considered in high-risk cardiac surgery with prolonged ICU. In general, an oral or enteral nutritional intake shall be continued after surgery to reduce surgical stress, maintain physiological functional capacity, and facilitate postoperative functional recovery. Yet, adequate strategies are still needed for an early identification of these cardiac surgery patients with prolonged ICU stay. In addition, more research is warranted, to evaluate the effect of an intense nutrition support on functional outcomes in this cohort of critically ill patients. Considering the patients` perioperative inflammatory response, adequately designed studies are supported by smaller pilot studies and currently under way to evaluate the clinical significance of different anti-inflammatory strategies. Given the high number of cardiac surgical ICU patients, future studies should aim to contribute to the development of specific nutrition guidelines for cardiac surgery patients.

## Figures and Tables

**Table 1:** Current nutrition standard in cardiac surgery as reported by Rahman et al. [32].

Form of Nutrition	Percentage of Patients	Caloric Adequacy	Protein Adequacy
EN	78%	25.5%	24.9%
EN + PN	17%	32.4%	28.8%

**Table 2:** Important nutrition screening tools.

Tool	Parameters	Source
ASPEN Guideline	Risk assessment with validated Score (NRS 2002, NUTRIC Score) Insufficient energy intake Weight loss Loss of muscle mass and subcutaneous fat Local or generalized fluid accumulation Diminished functional status as measured by handgrip strength	[24,31,34]
ESPEN Guideline	Risk screening with validated score (NRS 2002, MUS, MNA…) BMI <18.5 kg/m^2^ Weight loss	[23,29]
MNA-SF	Reduced food intake in the past 3 months Involuntary weight loss in the past 3 months Mobility Psychological stress or acute disease in the past 3 months neuropsychological problems BMI or calf circumference	[35,36]
MST	Weight loss Decreased appetite	[37]
MUST	BMI Involuntary weight loss Acute disease effect No nutritional intake expected for >5 days	[38,39,40,41]
NUTRIC Score	Age Acute Physiology and Chronic Health Evaluation II (APACHE II) Score Sequential Organ Failure Assessment (SOFA) Score Number of comorbidities Days from hospital to ICU admission IL-6 (optional)	[42,43,44,45]
NRS-2002	BMI <20.5 kg/m^2^ Weight loss in the last 3 months Reduced dietary intake Severe illness	[41,46]
SGA	Medical history: nutrient intake, weight, gastrointestinal symptoms, functional capacity, metabolic requirement Physical examination: loss of muscle mass and body fat, fluid retention	[47,48,49,50]
SNAQ	Unintended weight loss Decreased appetite Nutrition supplementation or tube feeding in the last 3 months	[51]

**Table 3:** Possible outcome parameters for nutrition interventions [55].

Period of Illness	Possible Outcome Parameters
Acute illness	Nutrition tolerance
	Protein balance
	Muscle mass
	Muscle biopsies
Physical function	Handgrip strength
	Quadriceps strength
	6-min walk distance
	Timed up and go test
	4-m gait speed
Participation in life	Activities of daily living
	Clinical frailty score
Quality of life	Short Form 36
	EQ-5D

**Table 4:** Prospective observational cohort studies examining the gastrointestinal response to enteral nutrition in the presence of compromised hemodynamics by evaluating intestinal intolerance.

Author, Year	No. of Patients	Time to Start of EN	Mean Energy Delivery	Vasopressor or Inotropic Drugs	Intestinal Tolerance
Berger 2005, [80]	70	<72 h	1360 ± 620 kcal/day	Median 5 days	No serious GI complications Prokinetics used in 12.9%
Revelly 2001, [81]	9	12–16 h	1.1 ± 0.25 kcal/kg/h	dobutamine (mean 420 µg/min) and norepinephrine (6–30 µg/min)	Hemodynamic response No change in catecholamine requirement Significant increase of cardiac index Transient decrease of mean arterial pressure Enteral and metabolic response No gut distension or digestive ischemia Increase in plasma glucose, decrease in fatty acids, increase in plasma lactate
Kesek 2002, [82]	62	<72 h	Depended individually as calculated by REE	n.a. ^1^	Vomiting: 20% Diarrhea: none 58%; mild 18%; moderate 21%; severe 3% GRV^1^: none 47%, small 19%; moderate: 11%; large 23% Aspiration pneumonia: 11% Prokinetics used in GRV > 400 mL
Flordelís Lasierra 2015, [84]	37	n.a.	1228.4 kcal/day	3 drugs: 38% 4 drugs: 24% 4 drugs + mechanical assistance in 16%	EN-related complications: 62% no serious GI complications constipation 46%, 1 case ischemic colitis attributed to prior vascular disease

^1^ GRV: Gastric residual volume, REE: resting energy expenidure, n.a.: not available.

## Abbreviations

AF	Atrial Fibrillation
ASPEN	American Society for Parenteral and Enteral Nutrition
BMI	Body Mass Index
CABG	Coronary Artery Bypass Graft
CK-MB	Creatine Kinase (muscle/brain)
CPB	Cardiopulmonary Bypass
CRP	C-reative Protein
DHA	Docosahexaenoic Acid
EN	Enteral Nutrition
EPA	Eicosapentanoic Acid
ERAS	Enhanced Recovery After Surgery
ESPEN	European Society for Parenteral and Enteral Nutrition
FO	Fish Oil
GI	Gastrointestinal
GRV	Gastric Residual Volume
ICU	Intensive Care Unit
IL	Interleukin
I/R	Ischemia/Reperfusion
LE	Lipid Emulsions
LOS	Length of Stay
MST	Malnutrition Screening Tool
MUST	Malnutrition Universal Screening Tool
n.a.	not available
NFκB	nuclear factor kappa-light-chain enhancer of activated B cells
NRS 2002	Nutrition Risk Screening 2002
NUTRIC	Nutrition Risk in the Critically Ill
PEEP	Positive End-expiratory pressure
PCT	Procalcitonin
PN	Parenteral Nutrition
PUFA	Polyunsaturated Fatty Acids
RCT	Randomized Controlled Trial
SGA	Subjective Global Assessment
SNAQ	Short Nutrition Assessment Questionnaire
sPN	Supplemental Parenteral Nutrition
TNFα	Tumor Necrosis Factor α
